# Endothelial dysfunction in children with newly diagnosed Graves’ disease

**DOI:** 10.1007/s00431-023-04919-z

**Published:** 2023-04-06

**Authors:** Yasser Gamal, Ahlam Badawy, Ahmed M. Ali, Hekma Saad Farghaly, Kotb Abbass Metwalley, Noha Gaber, Momtaz Thabet Allam, Yasser Farouk

**Affiliations:** 1 Department of Pediatrics, Faculty of Medicine, Assiut University, Assiut, Egypt; 2 Department of Clinical Pathology, South Egypt Cancer Institute, Assiut University, Assiut, Egypt; 3 Department of Radiology, Faculty of Medicine, Assiut University, Assiut, Egypt

**Keywords:** Graves’ disease, Flow-mediated dilatation, Thyrotropin receptor antibodies, Von Willebrand factor

## Abstract

The most frequent cause of hyperthyroidism in children is Graves’ disease (GD). Vascular endothelium is a specific target of thyroid hormone. The purpose of this study is to assess flow-mediated dilatation (FMD)% and serum von Willebrand factor (vWF) levels in children with newly diagnosed GD to reflect the extent of endothelial dysfunction in those children. In this study, 40 children with newly discovered GD and 40 children who were healthy served as the control group. Both patients and controls had anthropometric assessment, as well as measurements of fasting lipids, glucose, insulin, high-sensitivity C-reactive protein (hs-CRP), TSH, and free thyroxine (FT4 and FT3), thyrotropin receptor antibodies TRAbs and vWF. Noninvasive ultrasound was utilized to quantify the carotid arteries’ intima-media thickness and the brachial artery’s FMD. Patients reported significantly reduced FMD response and greater vWF and hs-CRP levels compared to controls (*P* = 0.001 for each). In multivariate analysis, we reported that vWF was significantly correlated with TSH (OR 2.5, 95% CI 1.32–5.32, *P* = 0.001), FT3 (OR 3.4, 95% CI 1.45–3.55, *P* = 0.001), TRAb (OR 2.1, 95% CI 1.16–2.23, *P* = 0.01), and FMD% (OR 4.2, 95% CI 1.18–8.23, *P* = 0.001).

*  Conclusions*: Children with newly diagnosed GD have endothelial dysfunction, which is shown by impaired FMD and increased vWF. These findings support the idea that GD may need to be treated as soon as possible.

**Table:** 

**What is Known:** *• Graves' disease is the most common cause of hyperthyroidism in children.* *• vWF is a reliable marker for detection of vascular endothelial dysfunction.*	
**What is New:** *• Children with newly diagnosed Graves' disease may have endothelial dysfunction as reflected by impairment of FMD and raised vWF level.* *• Measurement of vWF level in children with newly diagnosed Graves' disease can be used for early detection of endothelial dysfunction.*

**What is Known:**

*• Graves' disease is the most common cause of hyperthyroidism in children.*

*• vWF is a reliable marker for detection of vascular endothelial dysfunction.*

**What is New:**

*• Children with newly diagnosed Graves' disease may have endothelial dysfunction as reflected by impairment of FMD and raised vWF level.*

*• Measurement of vWF level in children with newly diagnosed Graves' disease can be used for early detection of endothelial dysfunction.*

## Introduction

Thyroid autoimmune illness with organ-specific symptoms is called Graves’ disease (GD) [1]. It accounts for between 10 and 15% of thyroid illnesses in children under the age of 18 and is the most prevalent cause of hyperthyroidism in the pediatric population [2]. It peaks during adolescence [3]. The etiology of GD is heavily influenced by immunological response. Autoantibodies, also known as thyroid-stimulating hormone receptor antibodies (TRAbs), are made by the immune system and cause the thyroid gland to overproduce thyroid hormones [4].

Napoli et al. [5] and Cui and Cheng [6] demonstrated that vascular endothelium is a specific target of thyroid hormones (TH), which can increase the amount of circulating adhesion molecules and nitric oxide (NO) by activating endothelium and perhaps slowing down their metabolism.

Vascular endothelial cells produce a substantial glycoprotein known as von Willebrand factor (vWF). When the endothelial cells are harmed, vWF is released into the plasma and by far it is the most accepted one for measurement of vascular endothelial dysfunction [7]. This is because it responds more rapidly to changes in endothelial function than endothelin 1, NO, and others [8]. vWF levels are raised in a number of inflammatory diseases such rheumatoid arthritis and vasculitis and are also related to inflammation [9].

A decreased vasodilatory response is a hallmark of endothelial dysfunction (ED), which is defined by irregularities in the control of the vascular lumen [10]. It is due to imbalance between relaxing and vasoconstricting factors, and pro-inflammatory mediators. Although evaluation of circulating endothelial function markers, such as vWF, provides the basis for the diagnosis of ED [1], measurement of brachial artery (FMD) is a currently recognized noninvasive useful technique for the evaluation of such ED [11].

Independent of other cardiovascular factors such dyslipidemia, obesity, and atrial fibrillation, hyperthyroidism may be associated with atherosclerotic cardiovascular disease, according to recent adult research [12, 13]. Carotid intima medium thickness (CIMT) is important early marker of such atherosclerosis [12].

To the best of our knowledge, there is no current research addressing the extent of endothelial dysfunction in children with GD. In order to determine how severely impacted the endothelium of those children is, we analyzed serum vWF levels, brachial artery (FMD), and carotid artery intima-media thickness in children with newly diagnosed GD and their relationships with clinical and laboratory data.

## Patients and methods

### Patients

This is a cross-sectional case–control study. It included 40 children and adolescents (age range: 10–18 years) carried the diagnosis of GD (girls = 28; boys = 12). Before beginning medical care, they were all newly diagnosed. They were successively chosen during a 2-year period from the Children’s Hospital of Assiut University in Assiut, Egypt’s Pediatric Endocrinology Clinic. Patients with GD met the following criteria: the presence of clinical manifestations of hyperthyroidism, thyroid-stimulating hormone (TSH) levels were low, free thyroxine (FT4) and free triiodothyronine (FT3) levels were high, thyrotropin receptor antibody (TRAb) titers were high, and sonographic features showed (decreased inhomogeneous echogenicity and increased vascularization) [14]. Children with other medical, systemic, or autoimmune diseases were excluded from the study. Also, cases with Graves’ ophthalmopathy, toxic adenoma, toxic multinodular goiter, subclinical hyperthyroidism, or those who used drugs which may influence the cardiovascular parameters were excluded from the study. Forty euthyroid healthy children (13 males and 27 females) matched for age, gender, pubertal status, and socioeconomic status were also included as control subjects. They were all chosen from the Assiut Children’s University Hospital’s General Pediatric Outpatient Clinic in Assiut, Egypt. They were there for a standard check-up at the outpatient clinic. None of the control subjects had a history of thyroid disease in the past or in their families, and all had negative antithyroid antibodies. Before being enrolled in the study, each patient, control subject, or their legal guardians gave their informed consent.

### Methods

To look for symptoms of thyroid illness or dysfunction, each participant got a thorough physical examination. Height and weight measurements were taken anthropometrically. The following formula was used to determine the body mass index (BMI): BMI equals weight (kg) × height (m)^2^. Standard deviation scores (SDSs) were used to express BMI in order to standardize for age and sex [15] using the Egyptian Growth Reference Data [16]. Pubertal development was assessed by determining Tanner stage [17]. Blood pressure was recorded and expressed as SDSs to normalize for age and sex [18].

#### Vascular ultrasound studies

All vascular ultrasound studies were performed using the GE Loqic P9 ultrasound system (General Electric Medical Systems) with an L3-12 Broadband Linear (3 to 12 MHz linear-array transducer).

#### Carotid intima-media thickness measurement

The right and left carotid arteries were scanned using a preset, standardized scanning methodology to measure the intima-media thickness (CA-IMT) in all subjects [19]. The children were lying supine, the transducer was placed on the contralateral vessel at a rough angle of 45°, and it was placed around 1 to 2 cm in front of the carotid artery’s bifurcation to get images of the right and left common carotid arteries. To assess the IMT of the posterior wall near the conclusion of diastole, the pictures of each artery were further enlarged (at the onset of the QRS complex). The carotid artery’s lumen-intima and media-adventitia ultrasonography interfaces were used to determine the IMT, which was measured in millimeters [20]. Independent, trained readers who were blinded to the subject’s clinical information conducted the image analysis.

#### Evaluation of endothelial function

A protocol developed in accordance with the recommendations for ultrasonographic evaluation of FMD was utilized to assess endothelial function [21]. Prior to the FMD examination, no high-fat foods, vitamin C supplements, stimulant-containing beverages, or strenuous exercise were permitted. After 30 min of relaxation and with the room temperature set to 20–25 C, the examinations were conducted on patients who had been fasting for at least 8 h. All tests were done early in the morning to eliminate circadian fluctuations. With the patient at rest, the brachial artery was imaged above the antecubital fossa, and its diameter was calculated from the B-mode ultrasound pictures (baseline brachial artery diameter). The next step was to apply a pneumatic tourniquet to the forearm and inflate it to a pressure of 250 mm Hg for 4.5 min before releasing it to cause reactive hyperemia. Using ultrasonic calipers, a second scan of the vessel’s diameter was carried out manually at a predetermined distance (maximal brachial artery diameter). On a continuously recorded ECG, longitudinal pictures were scanned and taken (in millimeters) at end diastole incident with the R-wave [22]. An expert physician with demonstrated skill in the method conducted the examinations. A synchronized ECG was obtained throughout the procedure. It was determined and the flow-mediated dilatation (FMD) was represented as a percentage. FMD was determined using the following formula [23]: FMD = % mean diameter of the hyperemic flow-baseline diameter/baseline diameter.

### Laboratory evaluation

In order to measure the serum levels of total cholesterol (TC), triglycerides, high-density lipoprotein cholesterol (HDL-c), low-density lipoprotein cholesterol, glucose, and insulin, blood samples were taken after an overnight fast of at least 12 h. Standard enzymatic techniques were used to measure the levels of TC, TG, HDL-c, and LDL-c using reagents from Boehringer Mannheim GmbH (Germany) and a fully automated analyzer. The Friedewald equation was used to calculate the LDL concentration [24]. Using the homeostasis model assessment (HOMA-IR) equation, insulin resistance (IR) was estimated as follows: HOMA-IR is defined as the product of fasting insulin (U/mL) and fasting glucose (mmol/L), divided by 22.5 [25], using very sensitive immunometric tests (IMMULITE 2000 Third Generation, Diagnostic Products Corporation, Los Angeles, CA) to estimate the serum levels of TSH, FT4, and FT3. TSH, FT4, and FT3 reference ranges are as follows: 0.4–4.0 mU/L for TSH, 10.0–26.0 pmol/L for FT4, and 3.5–5.5 pmol/L for FT3. The high sensitivity C reactive protein (hs-CRP) enzyme immunoassay test (ELISA) kit was used to measure the serum level of hs-CRP (catalog no. E29-056; Immunospec Corp., Canoga Park, CA, USA). Immunoassays were used to measure the serum TRAbs (Microparticle Enzyme Immunoassay–MEIA, Abbott AxSYM). Enzyme-linked immunosorbent assay was used to measure vWF using a commercially available kit (Reads med. Prod. Inc., Westminster, Colorado, USA).

### Statistical analysis

Statistical Package for the Social Sciences version 18.0 was used for all statistical analyses (Statistical Package for the Social Sciences Inc., Chicago, IL, USA). The data are displayed as the mean and standard deviation. For parametric data, the Student’s *t*-test was used to identify between-group differences, and for non-parametric data, the Mann–Whitney *U* test. We evaluated the linear relationships between vWF levels and other factors using Spearman’s or Pearson’s correlation coefficients for data with a normal distribution (non-normally distributed data). The independent significant connection between vWF and demographic, clinical, and laboratory factors was found using multiple logistic regression analysis (presented as odds ratios (ORs), 95% CI). Values of *p*0.05 were regarded as statistically significant for all tests.

## Results

The anthropometric, biochemical, and hormonal traits of patients and healthy controls are listed in Table 1. In terms of age and gender distribution, patients and controls were comparable. In comparison to controls, patients had significantly lower BMI SDS (*P* = 0.01) and faster heart rates (*P* = 0.01). Serum levels of vWF were significantly higher in GD subjects compared with controls (*P* = 0.01). However, no significant difference was found in the total cholesterol, triglycerides, LDL or HDL cholesterol, and HOMA IR between patients and controls.

**Table 1:** Clinical and laboratory characteristics of the studied groups

**Characteristics**	**GD cases** **(*n*** **= 40)**	**Controls** **(*n*** **= 40)**	***P*-value**
**Female/male**	28/12	27/13	NS
**Age(years)**	15.1 ± 3.1	14.2 ± 3.3	NS
**BMI SDS**	−0.32 ± 1.09	0.38 ± 2.43	0.01
**Heart rate; beat per minute**	116 ± 14	94 ± 8	0.01
**Systolic BP SDS**	0.68 ± 04	0.64 ± 0.3	NS
**Diastolic BP SDS**	0.38 ± 0.2	0.34 ± 0.07	NS
**TSH (mIU/ml)**	0.044 ± 0.03	2.95 ± 0.8	0.001
**FT4 (pmol/l)**	37.8 ± 9.3	14.43 ± 2.54	0.001
**FT3 (pmol/l)**	16.4 ± 4.4	4.21 ± 2.3	0.001
**Total cholesterol (mg/dl)**	143.2 ± 23.5	150.5 ± 15.1	NS
**Triglycerides (mg/dl)**	113.2 ± 19.1	115.1 ± 14.3	NS
**LDL-c (mg/dl)**	72.5 ± 65.2	75.6 ± 13.3	NS
**HDL-c (mg/dl)**	46.1 ± 7.8	43.9 ± 5.6	NS
**Fasting blood glucose (mg/dl)**	92.4 ± 9.8	88.6 ± 10.9	NS
**Fasting insulin (lU/mL)**	7.2 ± 2.2	8.6 ± 2.3	NS
**HOMA-IR**	1.6 ± 0.9	1.7 ± 0.8	NS
**hs-CRP (mg/L)**	329 ± 20.5	67.9 ± 12.8	0.001
**vWF (ng/ml)**	45.32 ± 11.5	6.2 ± 1.2	0.001

Data are means ± standard deviation (SD)

*BMI-SDS* body mass index standard deviation score, *TSH* thyroid-stimulating hormone, *FT4* free thyroxine, *FT3* free triiodothyronine, *TRAbs* thyrotropin-stimulating hormone receptor antibodies, high-sensitivity C-reactive protein, *HOMA-IR* homeostasis model assessment, *HDL-c* high-density lipoprotein cholesterol, *LDL-c* low-density lipoprotein cholesterol, *vWF* von Willebrand factor, *NS* non-significant

**Fig. 1:**
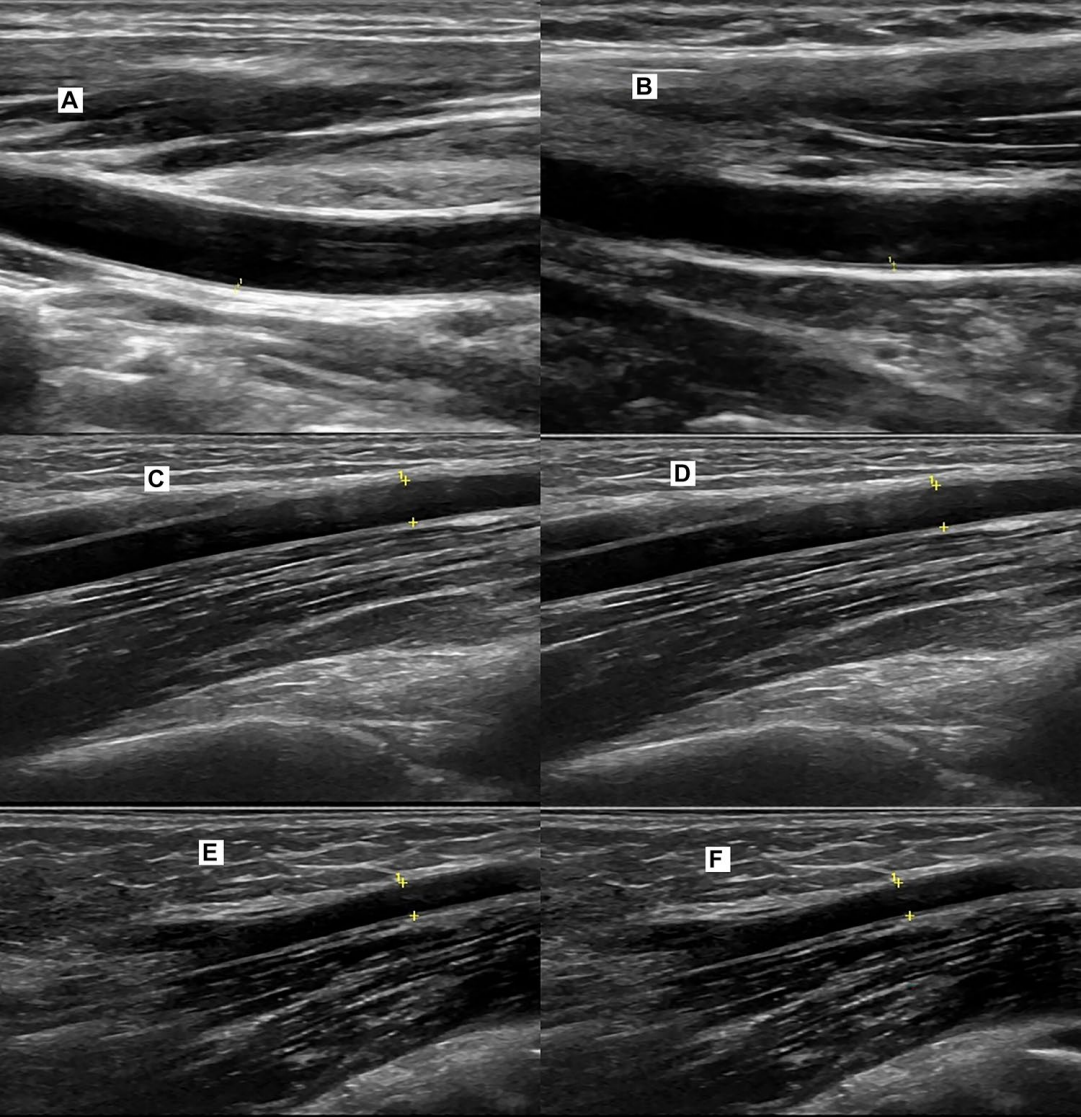
These images compare the CIM-T and FMD between control (female child 16 years) and another with Grave’s disease (female child 16 years old). **A** CIM-T in control and **B** in patient where it measures 0.5 mm with no significant differences. **C** Brachial artery diameter in control 18 mm and **D** FMD response to pneumatic inflation of tourniquet where it increases to 20 mm. **E** Brachial artery diameter in patient with Grave’s disease 19 mm and **F** FMD response to pneumatic inflation of tourniquet where it shows no significant change in brachial artery diameter

The CA-IMT and peak FMD characteristics of the studied groups are reported in Table 2. There was no significant difference between patients and control subjects as regards CA-IMT. However, compared to control subjects, patients had significantly lower peak FMD response (*P* = 0.001) (Fig. 1).

**Table 2:** CA-IMT, and FMD % values in the studied groups

	**GD cases** **(*n* = 40)**	**Controls** **(*n* = 40)**	
**CA-IMT, mm**	0.43 ± 0.07 ns	0.43 ± 0.086	NS
**FMD, %**	5.42 ± 2.15	11.42 ± 2.28	0.001

Data are expressed as mean ± SD

*CA-IMT* carotid intima-media thickness, *FMD* flow-mediated dilation, *ns* non-significant

The correlations between vWF and clinical and biochemical parameters among children with newly diagnosed GD are listed in Tables 3 and 4. We noted negative correlations between vWF, with TSH (−0.398, *P* = 0.01), and FMD % (−0.517, *P* = 0.001). There were positive correlations of vWF with FT3 (+ 0.626, *P* = 0.01), and TRAb (+ 0.456, *P* = 0.001). In multivariate analysis, we reported that vWF was significantly correlated with TSH (OR 2.5, 95% CI 1.32–5.32, *P* = 0.001), FT3 (OR 3.4, 95% CI 1.45–3.55, *P* = 0.001), TRAb (OR 2.1, 95% CI 1.16–2.23, *P* = 0.01), and FMD% (OR 4.2, 95% CI 1.18–8.23, *P* = 0.001).

**Table 3:** Correlation between vWF and various confounding variable among GD cases (*n* = 40)

Confounding variables	*r* value	*P* value
Age	0.164	NS
SDS-BMI	−0.50	NS
SBP (mmHg)	0.163	NS
DBP (mmHg)	0.142	NS
Total cholesterol (mg/dl)	0.171	NS
Triglycerides (mg/dl)	0.133	NS
HDL-c (mg/dl)	0.111	NS
LDL-c (mg/dl)	0.081	NS
Insulin (lU/mL)	0.82	NS
HOMA–IR	0.117	NS
hs-CRP (mg/L)	0.426	0.001
TSH (mIU/ml)	−0.534	0.001
FT4 (pmol/l)	0.091	NS
FT3 (pmol/l)	0.626	0.001
TRAb (IU/L)	0.456	0.001
CA-IMT (mm)	0.135	NS
FMD %	−0.517	0.001

*SDS-BMI* standard deviation scores of body mass index, *SBP* systolic blood pressure, *DBP* diastolic blood pressure, *HOMA-IR* the homeostasis model assessment of insulin resistance, *HDL-c* high-density lipoprotein cholesterol, *CA-IMT* carotid intima media thickness, *hs-CRP* high-sensitivity c reactive protein, *FMD* flow-mediated dilation, *TRAb* thyrotropin receptor antibodies, *TSH* thyroid-stimulating hormone, *FMD* flow-mediated dilation, *ns* non-significant

**Table 4:** Multivariate logistic regression analysis between vWF % and various variables in children with GD

**Confounding variables**	**Odds ratio**	95% confidence interval (CI)
TSH (mIU/ml)	2.5**	1.32–5.32
FT3 (pmol/l)	3.4**	1.45–3.55
TRAb( IU/L)	2.1**	1.16–2.23
FMD%	4.2***	1.18–8.23

*FMD* flow-mediated dilation, *CI* confidence interval, *TRAb* thyrotropin receptor antibodies, *TSH* thyroid-stimulating hormone

**Significance: 0.01; ***Significance: 0.001

## Discussion

In the current study, we observed that endothelial dysfunction occurs in children with Graves’ illness as evidenced by FMD impairment and elevated vWF. In view of recent research, thyroid hormones directly affect the synthesis of many hemostatic parameters, which results in most of the coagulation or fibrinolytic problems linked to thyroid dysfunction [26]. vWF is one of these substances that is produced by endothelial cell. Increased levels of vWF have been demonstrated to indicate ED. It was found to be elevated in both subclinical [27] and overt hyperthyroid adult groups [28].

This study, to our knowledge, is the first to show elevated vWF associated with reduced FMD as a marker of endothelial dysfunction in youngsters with newly diagnosed GD. Children with GD had significantly greater levels of vWF in the current study as compared to control children. Moreover, vWF was found to have positive correlation with FT3, while negative correlation with TSH remained significant after multiple regression analysis. This is in line with research by Van Zaane et al. [29] who showed that giving thyroid hormone to healthy adult volunteers dramatically raised plasma levels of vVF. Moreover, Ordookhani and Burman [30] reported that vWF is increased in adults with overt hyperthyroidism, where thyroid hormone excess stimulates thyroid receptors on endothelial cell and increase vWF level by influencing vVF synthesis through altering the expression of their genes.

Additionally, Popawska-Kita et al. [1] discovered that the vWF level in the hyperthyroid group was substantially higher than that in the euthyroid control group, while vWF did not correlate with FT3 and FT4, with only a substantial negative correlation between TSH and vWF.

However, Ellervik et al. [31] concluded that reduced and enhanced VWF synthesis may be linked to elevated TSH and fT4, respectively. Popławska-Kita et al. [1] tested the vWF and fibrinogen in adult hyperthyroidism and stated that vWF metabolites have the potential to harm and malfunction the endothelium. These findings imply a condition of relative hypercoagulability in patients with overt and subclinical hyperthyroidism. This condition may raise the risk of thromboembolism in those patients. Recently, Yu et al. [8] have tested for endothelial dysfunction in hyperthyroid rats and found that they had significantly higher values for vWF, TM, NO, ET-1, and P-selectin than control with the electron microscope showed a certain degree of endothelial injury of their abdominal aorta.

In the present work, we showed a substantial positive correlation between vWF and serum TRAbs levels, which remained significant following regression analysis and suggested a direct causative link between TRAbs and vWF. TRAbs which are involved in the pathogenesis of GD are associated with immune and inflammatory activities which induce increased vWF. This is in accordance with Coban et al. [32] study on subclinical hyperthyroid adults. So, it is suggested that elevated vWf concentrations are dependent not only on excess thyroid hormones but also on autoimmune factors. So, in addition to thyroid hormones and autoantibodies, measurement of vWF levels in children with newly diagnosed GD may be relevant as a clinical diagnostic for disease activity. However, it is crucial to note that neither our study nor the E Coban study was able to distinguish between the impact of autoimmunity and excessive thyroid hormone on the elevation of vWf.

CRP is a crucial component of low-grade artery wall inflammation, which is regarded to be the prelude to the development of atherosclerotic plaques, which happen just before structural changes. The effect of hyperthyroidism on CRP is still debatable [1]. Children with GD in this study had significantly greater circulating levels of hs CRP than did control children. These results are in line with those of Savas et al. [33] who found that adult GD patients had higher levels of systemic inflammation. Furthermore, we found that hs CRP levels were positively correlated with vWF levels. Increased production of these inflammatory markers could exacerbate endothelial dysfunction and confirm earlier findings linking inflammation to an increased risk of cardiovascular disease [34].

A 5-min period of distal limb ischemia causes the conduit vasodilator response of the brachial artery, known as FMD. The FMD process is endothelium dependent when carried out properly. Interventions that enhance endothelial function and FMD lower cardiovascular mortality and morbidity. Brachial FMD responses closely correlate with coronary endothelial function and predict cardiovascular end points [35]. According to this study, children with GD had significantly reduced brachial artery FMD% than matched controls, suggesting that endothelial function was compromised in these individuals. Moreover, FMD% correlated negatively and significantly with vWF that remained significant after regression analysis suggesting that endothelial dysfunction and endothelial activation are closely related to each other with established endothelial cell abnormalities in those children with GD. In this regard, a cohort of hypertensive and healthy adults according to Felmeden et al. [36] revealed a strong negative association between FMD% and vWF. Despite being an approved method for measuring endothelial function, FMD% is labor-intensive, is highly observer-dependent, and requires specific tools and technical expertise. Measurement of vWF levels may be a more practical diagnostic for the evaluation of endothelial abnormalities in such patients because vWF is a particular, persistent, circulating product of the endothelial cells and because of the tight link between it and FMD.

Endothelial dysfunction is thought to be an early and essential sign of atherosclerotic disease, and it can appear in the first 10 years of life35. The clinical end points for cardiovascular illnesses, such as acute myocardial infarction and cerebrovascular events, are risk assessed using the CA-IMT as a surrogate marker of atherosclerosis. Despite our findings of FMD abnormalities in such situations, there was no significant difference in CA-IMT between the patient group and the control group in this study. However, Bilir et al. [12] and Vöwas no significant difference in CAlzke et al. [37] found that adult Graves’ hyperthyroidism is associated with significantly increased CIMT, positive relation with advanced age. In addition, they found that treatment of Graves’ hyperthyroidism with propylthiouracil decreases the CA-IMT. Such discrepancy from our results can be explained by young age of our study population and the short-term onset of Graves’ disease in them. In this context, Babar et al. [38] reported no difference in CA-IMT in a cohort of a children with type 1 diabetes mellitus compared to controls in spite of low FMD%. This can be explained by the fact that although FMD is a dynamic measure that represents the impact of both acute and chronic factors on endothelial function, CA-IMT is a measure of structural long-term alterations [39]. Therefore, impaired FMD in our patients can only be an early stage before any CA-IMT alteration. It is crucial to be able to spot these early alterations in vascular function in kids with Graves’ disease because endothelial dysfunction has been demonstrated to be independently related with the development of alterations in CA-IMT over time [40].

Notably, no medication that successfully target and repair damaged vascular endothelial cells have been approved for clinical use in the treatment of individuals with ED. Nonetheless, exercise and lifestyle changes are extremely important in the therapy of patients with GD and elevated vWF. Physical activity has been shown to have positive effects on the treatment of ED, including an increase in the bioavailability of nitric oxide (NO), a decrease in oxidative stress, an increase in the number of circulating endothelial progenitor cells, a suppression of pro-inflammatory cytokines, and increase in the activity of the AMPK SIRT1 and miR-126. Two important exercise-protection sensors (SIRT1 and miR-126) could serve as brand-new therapeutic targets for the treatment of endothelial injury [41].

## Limitations

This study’s main constraints include its cross-sectional design and small sample size, which make cautious interpretation of the findings necessary. Another limitation is the lack of information regarding the length of thyroid dysfunction before a verified diagnosis. Additionally, because patients self-referred for care, there may be selection bias when extrapolating the clinical traits of this cohort to the overall population with GD. Also follow-up assessment of the biomarkers was mandatory in this study; however, some of our patients were missed follow-up visit, due to either long distance from their residence or poor compliance so the remaining number was too small to be analyzed.

## Conclusions

In conclusion, the present study showed that children with Graves’ disease experience endothelial dysfunction, as demonstrated by impairment of FMD and raised vWF. So it is advised to measure the plasma levels of vWF in all children with overt hyperthyroidism in order to identify extent of endothelial dysfunction with early diagnosis and follow-up so as to reduce risk of cardiovascular and thromboembolic complications.

## Data Availability

The datasets used and/or analyzed during the current study are available from the corresponding author upon reasonable request.

## References

[1] Popławska-Kita A, Szelachowska M, Modzelewska A, Siewko K, Dzięcioł J, Klimiuk PA, Górska M (2013) Endothelial dysfunction in Graves’ disease. Adv Med Sci 58(1):31–37· 10.2478/v10039-012-0047-1 10.2478/v10039-012-0047-1 23612675

[2] Zimmerman D, Lteif AN (1998). Thyrotoxicosis in children. Endocrinol Metab Clin N Am.

[3] Leger J, Kaguelidou F (2014). Graves’ disease in children. Best Pract Res Clin Endocrinol Metab.

[4] Cappelli C, Gandossi E, Castellano M, Pizzocaro C, Agosti B, Delbarba A et al (2007) Prognostic value of thyrotropin receptor antibodies (TRAb) in Graves’ disease: a 120 months prospective study. Endocr J (5):713–720 10.1507/endocrj.k06-069 17675761

[5] Napoli R, Biondi B, Guardasole V, Matarazzo M, Pardo F, Angelini V, Fazio S, Saccà L (2001). Impact of hyperthyroidism and its correction on vascular reactivity in humans. Clinical Trial Circulation.

[6] Cui L, Cheng G (2017) The research progress of the effect of nitric oxide in vascular endothelial injury induced by hypothyroidism. Chin Mod Doc

[7] Horvath B, Hegedus D, Szapary L, Marton Z, Alexy T, Koltai K, Czopf L, Wittmann I, Juricskay I, Toth K, Kesmarky G (2004). Measurement of von Willebrand factor as the marker of endothelial dysfunction in vascular diseases. Exp Clin Cardiol.

[8] Yu T, Jing M. , Gao Y et al (2020) Study on the relationship between hyperthyroidism and vascular endothelial cell damage. Sci Rep 10(6992). 10.1038/s41598-020-62796-0 10.1038/s41598-020-62796-0 PMC7181772 32332761

[9] Gurol G, Ciftci IH, Harman H, Karakece E, Kamanli A, Tekeoglu I (2015). Roles of claudin-5 and von Willebrand factor in patients with rheumatoid arthritis. Int J Clin Exp Pathol.

[10] Landmesser U, Hornig B, Drexler H (2004) Endothelial function: a critical determinant in atherosclerosis? Circulation 109(21 Suppl 1):II27–33 10.1161/01.CIR.0000129501.88485.1f 15173060

[11] Wilk G, Osmenda G, Matusik P, Nowakowski D, Jasiewicz-Honkisz B, Ignacak A, Guzik TJ (2013). Endothelial function assessment in atherosclerosis: comparison of brachial artery flow-mediated vasodilation and peripheral arterial tonometry. Polish Archives of Internal Medicine.

[12] Bilir C, Gökosmanoglu F, Caliskan M, Cinemre H, Akdemir R et al (2012) Regression of the carotid intima media thickness by propylthiouracil therapy in Graves’ hyperthyroidism. Am J Med Sci 343(4):273–6. 10.1097/MAJ.0b013e31822a8284 10.1097/MAJ.0b013e31822a8284 21825964

[13] Wisnu W, Alwi I, Nafrialdi N, Harimurti K, GedeT., Widia S et al (2021) The differential effects of propylthiouracil and methimazole as Graves’ disease treatment on vascular atherosclerosis markers: a randomized clinical trial. Front Endocrinol (Lausanne)12:796194. 10.3389/fendo.2021.796194 10.3389/fendo.2021.796194 PMC8721229 34987480

[14] Weetman AP (2003). Grave’s disease 1835–2002. Horm Res.

[15] Rolland-Cachera MF, Cole TJ, Sempe M, Tichet J, Rossignol C, Charraud A (1991). Body mass index variations: centiles from birth to 87 years. Eur J Clin Nutr.

[16] Diabetes Endocrine Metabolism Pediatric Unit (2002) Cairo University Children’s Hospital: Egyptian growth curves. Available from http://www.dempuegypt.blogspot.com. Accessed 15 May 2021

[17] Marshall WA, Tanner JM (1970). Variations in the pattern of pubertal changes in boys. Arch Dis Child.

[18] Demir K, Konakçı E, Özkaya G, Kasap BD, Özen S, Aydın M, Darendeliler F (2019) New features for child metrics: further growth references and blood pressure calculations. J Clin Res Pediatr Endocrinol 10.4274/jcrpe.galenos.2019.2019.0127 PMC7291402 31475511

[19] Jarvisalo MJ, Jartti L, Näntö-Salonen K, Irjala K, Rönnemaa T, Hartiala JJ et al (2001) Increased aortic intima-media thickness: a marker of preclinical atherosclerosis in high-risk children. Circulation 104(24):2943–2947 10.1161/hc4901.100522 11739310

[20] Kanters SD, Algra A, van Leeuwen MS (1997). Banga JD : Reproducibility of in vivo carotid intima-media thickness measurements: a review. Stroke.

[21] Järvisalo MJ, Rönnemaa T, Volanen I (2002). Brachial artery dilatation responses in healthy children and adolescents. Am J Physiol Heart Circ Physiol.

[22] Kizhakekuttu TJ, Gutterman DD, Phillips SA (2010). Measuring FMD in the brachial artery: how important is QRS-gating?. J Appl Physiol.

[23] Thijssen DH, van Bemmel MM, Bullens LM, Dawson EA, Hopkins ND, Tinken TM, Black MA, Hopman MT, Cable NT, Green DJ (2008) The impact of baseline diameter on flow-mediated dilation differs in young and older humans. Am J Physiol Heart Circ Physiol 295(4):H1594–H1598 10.1152/ajpheart.00669.2008 PMC2593521 18708443

[24] Friedewald WT, Levy RI (1972). Fredrickson DS : Estimation of the concentration of low-density lipoprotein cholesterol in plasma, without use of the preparative ultracentrifuge. Clin Chem.

[25] Matthews DR, Hosker JP, Rudenski AS, Naylor BA, Treacher DF, Turner RC (1985). Homeostasis model assessment: insulin resistance and beta-cell function from fasting plasma glucose and insulin concentrations in man. Diabetologia.

[26] Mina A, Favaloro EJ, Koutts J (2007) Hemostatic dysfunction associated with endocrine disorders as a major risk factor and cause of human morbidity and mortality: a comprehensive meta-review. Semin Thromb Hemost 33(8):798–809 10.1055/s-2007-1000372 18175285

[27] Tamer I, Sargin M, Sargin H, Seker M, Babalik E, Tekce M, Yayla A (2005). The evaluation of left ventricular hypertrophy in hypertensive patients with subclinical hyperthyroidism. Endocr J.

[28] Li Y, Chen H, Tan J, Wang X, Liang H, Sun X (1998). Impaired release of tissue plasminogen activator from the endothelium in Graves’ disease - indicator of endothelial dysfunction and reduced fibrinolytic capacity. Eur J Clin Invest.

[29] van Zaane B, Squizzato A, Debeij J, Dekkers OM, Meijers JC, van Zanten AP, Brandjes DP (2011). Alterations in coagulation and fibrinolysis after levothyroxine exposure in healthy volunteers: a controlled randomized crossover study. J Thromb Haemost.

[30] Ordookhani A, Burman KD (2017). Hemostasis in overt and subclinical hyperthyroidism. International journal of endocrinology and metabolism.

[31] Ellervik C, Mora S, Kuś A, Åsvold B, Marouli E et al (2021) Effects of thyroid function on hemostasis, coagulation, and fibrinolysis: a mendelian randomization study. Thyroid 31(9). Mary Ann Liebert Inc. 10.1089/thy.2021.0055 10.1089/thy.2021.0055 PMC8558080 34210154

[32] Coban E, Aydemir M, Yazicioglu G, Ozdogan M (2006). Endothelial dysfunction in subjects with subclinical hyperthyroidism J Endocrinol Invest.

[33] Savas E, Sahin AZ, Aksoy SN, Tascan A, Sayıner ZA, Ozkaya M (2016) Serum levels of inflammatory markers in patients with thyroid dysfunction and their association with autoimmunity status. Int J Clin Exp Med 9(2):4485–4490

[34] Rewiuk K, Grodzicki T (2015) Correlations of C-reactive protein, von Willebrand factor, and carotid artery intima-media thickness with CHA2DS2-VASc in patients with acute atrial fibrillation. Polskie Archiwum Medycyny Wewnętrznej Pol Arch Int Med 125(11) 10.20452/pamw.3162 26463558

[35] Hopkins ND, Stratton G, Tinken TM, Ridgers ND (2011). Seasonal reduction physical activity and flow-mediated dilation in children. Med Sci Sports Exerc.

[36] Felmeden DC, Blann AD, Spencer CG, Beevers DG, Lip GY (2003). A comparison of flow-mediated dilatation and von Willebrand factor as markers of endothelial cell function in health and in hypertension: relationship to cardiovascular risk and effects of treatment A substudy of the Anglo-Scandinavian Cardiac Outcomes Trial. Blood Coag Fibrinol.

[37] Völzke H, Robinson DM, Schminke U et al (2004) Thyroid function and carotid wall thickness. J Clin Endocrinol Metab 89:2145–9 10.1210/jc.2003-031028 15126533

[38] Babar GS, Zidan H, Widlansky ME, Das E, Hoffmann RG, Daoud M, Alemzadeh R (2011). Impaired endothelial function in preadolescent children with type 1 diabetes. Diabetes Care.

[39] Halcox JP, Donald AE, Ellins E, Witte DR, Shipley MJ, Brunner EJ (2009). Endothelial function predicts progression of carotid intima- media thickness. Circulation.

[40] Froehlich H, Haas E et al (2014) Endothelial dysfunction and brachial intima-media thickness: long term cardiovascular risk with claudication related to peripheral arterial disease: a prospective analysis. PLoS One 9(4):e93357 10.1371/journal.pone.0093357 PMC3989175 24740106

[41] Gao J, Pan X, Li G, Chatterjee E, Xiao J (2022). Physical exercise protects against endothelial dysfunction in cardiovascular and metabolic diseases. J Cardiovasc Transl Res.

